# Evaluation of a co-designed educational e-resource about oral health for community nurses: study protocol

**DOI:** 10.1186/s12912-023-01268-y

**Published:** 2023-04-03

**Authors:** Patrick Stark, Gerry McKenna, Christine Brown Wilson, Georgios Tsakos, Paul Brocklehurst, Caroline Lappin, Barry Quinn, Nicola Holland, Gary Mitchell

**Affiliations:** 1 School of Nursing and Midwifery, Queen’s University Belfast, Belfast, Northern Ireland; 2 Centre for Public Health, Queen’s University Belfast, Belfast, Northern Ireland; 3 Department of Epidemiology and Public Health, University College London, London, UK; 4 School of Health Sciences, Bangor University, Bangor, UK; 5 Department of Health, Castle Buildings, Stormont, Belfast, Northern Ireland

**Keywords:** Oral health, Oral Healthcare, Older people, Nursing, Community nursing, District nursing, Home Healthcare, Home Health Care Services, Workforce

## Abstract

**Background:**

Oral health is a crucial aspect of health and wellbeing for older people. Poor oral health has been found to significantly increase the risk of chronic health conditions and poor quality of life for older people. Nurses practicing in the community are well-placed to provide oral health care to older people in their own homes, yet there has been little research in this field to develop accessible support for them to do so. Previous literature, reviewed in an earlier phase of this work, revealed that there has, historically, been a paucity of oral health care education for nurses and very few educational resources have been developed in this field.

**Methods:**

This study will evaluate an educational e-resource which has been co-designed by service users, carers and clinicians. In the first phase of research, evidence of promise will be evaluated by analysing quantitative data on community nurses’ oral health attitudes and self-efficacy for oral health assessments of older people. In the second phase of research, facilitators and barriers to community nurses’ provision of oral health care to older people and the acceptability of the educational e-resource will be evaluated.

**Discussion:**

This research will investigate the potential of an educational e-resource to improve community nurses’ capabilities to deliver oral health care to older people in their own homes. This research will inform both future intervention design and understanding of community nurses’ knowledge and attitudes about oral health care. Facilitators and barriers to provision of this care for older people will also be explored.

**Supplementary Information:**

The online version contains supplementary material available at 10.1186/s12912-023-01268-y.

## Background

Almost half of the world’s population experience oral diseases. A subgroup of this global population that are at high risk of oral health disease are those who are older [1, 2]. Older people are more likely to experience oral health complications in the form of tooth loss, tooth decay, gum disease, dry mouth and cancer of the mouth [1].

The negative impact of poor oral conditions on the quality of life of older adults is an important public health issue, which must be addressed. As well as causing pain and making it difficult to speak, eat and take medication, poor oral health is linked to conditions such as malnutrition and aspiration pneumonia. Within the United Kingdom (UK), at least 1.8 million people aged 65 and over could have an urgent dental condition such as dental pain, oral sepsis, or extensive decay in untreated teeth. By 2040, this number is expected to increase by more than 50% [2].

There are major challenges in translating this knowledge into action for the oral health of older people. There is also a plethora of evidence that suggests that older people, living with complex needs, struggle to access dental services [3]. The needs for oral care are highest among those older people who live with complex comorbidities (i.e., dementia, heart failure, COPD, frailty, and diabetes) and are unable to regularly visit their dentist [4]. Education and continuous training must ensure that oral health care providers have skills in and a profound understanding of the biomedical and psychosocial aspects of care for older people [5].

The demand for dental services will increase as people continue to grow older and healthcare professional education programmes must evaluate their curricula to reflect older people’s changing needs in relation to oral care. This project will seek to provide front-line district and community nurses with a digital e-resource, which will support the assessment and management of oral care for older adults living at home in the community. This patient group is currently particularly underserved as they may no longer access services from General Dental Practitioners (family dentists) whilst Community Dental Services struggle to provide domiciliary care [6]. Promotion of optimal oral health can indeed improve and maintain the overall health and wellbeing of an older person. Therefore, ensuring basic oral health care for patients in community nursing care is unquestionably a professional responsibility for nurses as part of a multidisciplinary approach [7].

### Evidence base

This project draws on several evidence-based inputs, carried out in earlier phases of this research, which have been formative in its design. Firstly, a grounded theory study was conducted to build a theory of current barriers and facilitators to community nurses’ delivery of oral health care [8]. This study revealed significant challenges in terms of professional development around oral health care and a perception that it was beyond the scope or role of community nurses to deliver this care. Building on this grounded theory, the present study will actively seek to educate community nurses about best practice, and about how it can fit within their current role.

Secondly, a scoping review was carried out and published which synthesised previous literature on oral health interventions in community nursing settings [9]. The review found a clear gap in the research around interventions designed to be used by community nurses. It also found that in the small amount of previous literature that exists, there is evidence that such interventions can be beneficial to patient outcomes and to the development of nurses’ skill in this area. Findings from this review also suggested that any future intervention should make use of a co-design approach and consider the complex setting of nursing practice in the community, as key barriers to implementation were time pressure and professional support.

These two evidence-bases are the first inputs which will be used to develop a logic model at the completion of this project, mapping evidence-based programme inputs, activities/outputs, potential proximal and distal outcomes and facilitators and barriers to implementation [10]. These inputs were shared with the co-design team for the educational e-resource and were formative in ensuring that it has as much evidence informing its design as is available, making use of both the practical realities as shared by nurses and the synthesis of previous empirical research.

### Aim

The aim of this project is to evaluate a recently co-designed e-resource about oral health for community nurses in Northern Ireland.

The objectives of this study are to:

i. Evaluate this e-resource amongst community nurses who provide care or support older people in their own homes.
ii. Explore if this intervention affects community nursing attitude to oral health and self-efficacy for oral health assessment.
iii. Explore acceptability of this e-resource amongst community nurses.

## Methods

### Design

This study has been designed to run in two consecutive phases. In Phase 1, approx. n = 100 community nurses will use the educational e-resource, and changes in oral health attitudes and self-efficacy of these community nurses will be assessed using quantitative measures administered before and after using the e-resource. In Phase 2, a smaller number of these nurses (n = 12) will participate in qualitative data collection via semi-structured interviews, to explore facilitators and barriers to providing oral health care to older people in their own homes and to explore acceptability of using the e-resource.

### Educational e-resource

The educational e-resource is currently under development following co-design with service users, carers and clinicians. Its design has also been informed by a scoping review of oral healthcare interventions in community nursing settings and qualitative field work [8, 9]. The e-resource will be freely accessible via a HTML5 web application with a supporting website. The intervention will work on any device through a web browser and will have some functionality offline. The educational e-resource will provide community nurses with:

i. Evidence based health promotion strategies to support prevention of oral health complications in older people living in community settings.
ii. Access and information to appropriate tools to support promotion of optimum practice pertaining to oral health.
iii. Information about who to contact if an older person presents with oral health problems and how to provide immediate supportive care to manage oral health problems while awaiting healthcare professional intervention.

This e-resource will accommodate videos, audio, pictures, recommended assessment tools and external links to guidance for community nurses as appropriate.

### Ethics and consent

The study has been reviewed and approved by Queen’s University Belfast, Medical Health & Life Sciences Ethics Committee (MHLS 22_65). Online informed consent will be obtained from all questionnaire participants and written informed consent will be obtained from all interviewed participants. All methods will be performed in accordance with the Declaration of Helsinki.

### Recruitment – phase 1

We are aiming to evaluate the digital intervention amongst approx. n = 100 community nurses in Northern Ireland. The research team will be supported to recruit community nurses (within Northern Ireland only) by the Queen’s Nursing Institute (QNI) and the Royal College of Nursing (RCN). These organisations will contact their community nurse members in Northern Ireland via their monthly newsletter (via email). These organisations regularly disseminate information about research to their members (which collectively will reach 2,000–3,000 eligible participants).

Potential participants who click on the link will be taken to an online information sheet and online consent form at the beginning of the e-resource. If they choose to participate, they will form the sample for quantitative data collection in Phase 1 and be given immediate access to the e-resource at this time. Details on how to complete the pre and post tests will be embedded within the e-resource. Users will be directed to complete the pre-test immediately after providing consent, i.e., before they access the e-resource. The post-test will automatically be generated at the end of the e-resource. If a user does not complete the e-resource, we will not capture a post-test. The e-resource will take approximately 30–45 min to complete and automatically save the progress of the user.

At the conclusion of the post-test, participants will also be asked if they would like to be contacted for qualitative data collection in Phase 2. They will provide their preferred contact details and will be followed up with by the research team.

In the interests of fairness, if a participant clicks on the link and wishes to use the e-resource, but does not consent to the research, they will still be permitted to access the educational resource but will not be part of the study.

### Recruitment – phase 2

Phase two of the research will take place after the completion of phase one and will focus on acceptability of this e-resource amongst community nurses. The research team will conduct individual semi-structured interviews with community nurses who used the digital resource. Interviews will take place via Microsoft teams, telephone or face-to-face depending on the preference of the participant. We are aiming to recruit 12 community nurses. The precise number of recruits may increase or decrease depending on when data saturation occurs [9]. All participants will be required to provide written consent prior to the commencement of online, telephone or face-to-face interview.

### Data collection instruments – phase 1

Phase 1 participants will be given a previously developed DELPHI survey to measure attitudes of oral health care for older people used previously in care settings [11]. This will be delivered to participants before and after using the oral health education resource. This is to measure possible attitude change about oral health amongst community nurses. Based on the team’s previous experience of using these questionnaires, we anticipate that they will take approximately ten minutes to complete. We will also administer a self-efficacy measure, assessing self-efficacy for providing oral health assessments and communicating with older people about their oral health. Due to the nature of this study, and the lack of empirical investigation of self-efficacy in this field, it was not possible to use an existing measure. Self-efficacy is domain specific, and the research team have developed a 14-item scale in accordance with Bandura’s guidelines [12].

### Data collection process – phase 2

Semi-structured interviews will be undertaken using a co-designed interview guide designed to explore community nurses’ experiences of providing oral health care to older people in their own homes and their experiences of using the educational e-resource. Audio recordings will be made of these interviews to facilitate transcription and analysis. The second phase of this study will adhere to the consolidated criteria for reporting qualitative research (COREQ) checklist [13] for methodological rigour. Please see supplementary file 1 for further information.

### Setting & participants

In phase one, all data collection will take place through the online surveys at the participants’ convenience. In phase two, all data collection will take place away from a health and social care setting while community/district nurses are ‘off-duty’. The interviews will take place online using Microsoft Teams. The specific inclusion and exclusion criteria for both phases are noted in Table 1.

**Table 1:** Inclusion and Exclusion Criteria for Phase 1 & 2 of Study

Inclusion Criteria: Phase 1 & 2	Exclusion Criteria: Phase 1 & 2
Community nurses in Northern Ireland who have accessed the e-resource.	Community nurses in Northern Ireland who have not accessed the digital intervention. Nurses from other areas of practice who do not work in the community. Any nurse practising outside of Northern Ireland

### Analysis

In phase one, all data will be analysed in SPSS. The research team will use descriptive statistics and two paired sample t-tests to determine if there is a statistically significant difference between pre-test and post-test scores for the oral health DELPHI questionnaire and the self-efficacy questionnaire. Reliability of the scales will also be investigated using reliability analysis via Cronbach’s alpha. In phase two, all interviews will be transcribed verbatim and uploaded to NVivo11 management software. Qualitative analysis will be conducted using thematic analysis [14]. The combination of these two analytical approaches will address aims (ii); Explore if this intervention affects community nursing attitude to oral health and self-efficacy for oral health assessment and (iii); Explore acceptability of this e-resource amongst community nurses.

## Discussion

As previous empirical evidence has demonstrated, the oral health of older people is an underserved area of research and practice and this study has the potential to make a novel contribution to practice [8, 9, 15–17]. Home care nursing, or community nursing as it is referred to in the United Kingdom, is an important area for research. Community nurses require support in providing oral health care to older people who may experience plaque, caries, xerostomia, chewing problems and swallowing problems [15–17]. Despite this, community nurses are often uncertain about their role in provision of oral health care and existing continuing professional development opportunities appear to be limited within nursing education [8, 18, 19].

Following the evaluation of this study, the e-resource will be freely available to all. The research team is hopeful that this e-resource will support the development in knowledge, improve community nursing self-efficacy in the area of oral healthcare and improve patient care.

## Electronic supplementary material

Below is the link to the electronic supplementary material.

Supplementary Material 1

## Data Availability

Data sharing is not applicable to this article as no datasets were generated or analysed during the production of this study protocol.
