# Supplementary material for: Evaluation of a co-designed educational e-resource about oral health for community nurses: study protocol
Source: BMC Nurs. 2023 Apr 3;22:94. doi: 10.1186/s12912-023-01268-y (PMC10071717; doi:10.1186/s12912-023-01268-y)
Supplement: Supplementary file 1 — Supplementary Material 1 [file 12912_2023_1268_MOESM1_ESM.docx]

**Supplementary File 1**

Consolidated criteria for reporting qualitative studies (COREQ): 32-item checklist.

| **No** | **Item** | **Guide questions/description** |
| --- | --- | --- |
| **Domain 1: Research team and reflexivity** |  |  |
| Personal Characteristics |  |  |
| 1. | Interviewer/facilitator | All interviews will be conducted by Dr Gary Mitchell (GM). |
| 2. | Credentials | GM has a PhD and has expertise in qualitative research methods and qualitative data collection (including semi-structured interviewing). |
| 3. | Occupation | GM is a senior lecturer at Queen’s University Belfast in Northern Ireland. |
| 4. | Gender | GM is male. |
| 5. | Experience and training | GM is a registered nurse with considerable clinical experience in community healthcare and care of older people. He has topic expertise and holds the title of Queen’s Nurse. |
| Relationship with participants |  |  |
| 6. | Relationship established | The sample has yet to be recruited, however it is anticipated that community nurses will not be known to GM. |
| 7. | Participant knowledge of the interviewer | Participants will be aware of who is conducting the interview as this is detailed in the information sheet. |
| 8. | Interviewer characteristics | GM is a registered nurse with clinical expertise in the topic area. GM also has a strong awareness in the context (e.g., delivering care to older people living at home). Prior to the interviews, GM has been involved in a scoping review of the literature and an exploratory study on the same topic area. |
| **Domain 2: study design** |  |  |
| Theoretical framework |  |  |
| 9. | Methodological orientation and Theory | The qualitative component of this study will use interpretivist approach that focuses on the acceptability of the digital intervention. |
| Participant selection |  |  |
| 10. | Sampling | Convenience sampling will be used in the form of adverts in newsletters which are sent to the members of the Queen’s Nursing Institute (QNI) and the Royal College of Nursing (RCN). |
| 11. | Method of approach | Participants will be approached via email only. |
| 12. | Sample size | We are aiming to participate 12 community nurses in phase two. |
| 13. | Non-participation | Participants will be reminded that participating in this research will not affect their employment status or job performance and that participation is voluntary. |
| Setting |  |  |
| 14. | Setting of data collection | Data will be collected via online meeting, telephone or face-to-face depending on the participant’s preference. Data collection will not take place during a participant’s working time. |
| 15. | Presence of non-participants | There will be no non-participants present during the semi-structured interviews, only GM and the participant. |
| 16. | Description of sample | Participants will be registered nurses who provide care to people living in their own homes (e.g., community nurses, district nurses or home care nurses). They will also be nurses who have used the digital resource. |
| Data collection |  |  |
| 17. | Interview guide | The interview guide has been co-designed by experienced community nurses and oral health professionals. The interview guide has also been piloted with two community nurses. |
| 18. | Repeat interviews | It is not envisaged that repeat interviews will be carried out. |
| 19. | Audio/visual recording | All semi-structured interviews will be audio-recorded. |
| 20. | Field notes | No field notes will be collected during semi-structured interviews. |
| 21. | Duration | Semi-structured interviews are anticipated to last between 30-45 minutes. |
| 22. | Data saturation | Based on previous experience and research in the field, it is anticipated that approximately 12 participants will be needed to reach saturation. |
| 23. | Transcripts returned | 25% of transcripts (e.g., 3 out of 12) will be returned to participants to support member checking. |
| **Domain 3: analysis and findings** |  |  |
| Data analysis |  |  |
| 24. | Number of data coders | GM, PS & CBW will be the three authors responsible for coding the data from this study. |
| 25. | Description of the coding tree | All authors will be consulted in the development of the coding tree. |
| 26. | Derivation of themes | The authors will use thematic analysis to analyse themes. This will be led by GM, PS & CBW but will involve all members of the authorship team in the advanced stages. |
| 27. | Software | The authors will use NVivo Version 12 to manage the data. |
| 28. | Participant checking | A minimum of 25% of participants will be involved in member checking. |
| Reporting |  |  |
| 29. | Quotations presented | Direct quotations will be presented from participants in any publications, conference proceedings or funder report. Participant confidentiality will be maintained. |
| 30. | Data and findings consistent | The authors will ensure there is concordance between the data and findings that are presented. |
| 31. | Clarity of major themes | The authors will report all major themes as identified in this research. |
| 32. | Clarity of minor themes | The authors will report on any minor themes or deviant cases within their presentation of results. |
